# Segmenting OCT for detecting drug efficacy in CRAO

**DOI:** 10.1371/journal.pone.0242920

**Published:** 2020-12-11

**Authors:** Thomas R. Shearer, Thomas S. Hwang, Peter N. Steinkamp, Mitsuyoshi Azuma

**Affiliations:** 1 Department of Integrative Biomedical & Diagnostic Sciences, Oregon Health & Science University, Portland, OR, United States of America; 2 Department of Ophthalmology-OHSU, Portland, OR, United States of America; 3 Senju Laboratory of Ocular Sciences, Portland, OR, United States of America; Bascom Palmer Eye Institute, UNITED STATES

## Abstract

**Purpose:**

Thinning of the inner layers of the retina occurs in patients with central retinal artery occlusion (CRAO). The mechanism for such thinning may be partially due to proteolysis by a calcium-activated protease called calpain. Calpain inhibitor SNJ-1945 ameliorated the proteolysis in a past series of model experiments. The purposes of the present retrospective study were to: 1) use segmentation analysis of optical coherence tomography (OCT) images to mathematically model the loss of specific retinal layers in CRAO patients, and 2) predict the number of patients and days of observation needed for clinical trials of inhibitors against CRAO.

**Methods:**

A retrospective case control study was conducted by computer-aided search for CRAO (ICD10 H43.1) with at least one OCT procedure (CPT: 92134) in the OHSU Epic patient data base.

**Results:**

After initial swelling, thinning of the inner retinal layers, especially the ganglion cell (GCL) layer followed exponential decay curves. Using sample size statistics and GCL thickness as a marker in a 30-day clinical trial, 19 eyes/group could theoretically detect a 20% beneficial effect of an inhibitor against CRAO. Other markers, such as the whole retinal thickness and combined inner layers could also be used as less-specific markers.

**Conclusions:**

Using thickness changes in the GCL layer to monitor the efficacy of potential inhibitors against CRAO is practical in human trials requiring a reasonable number of patients and relatively short trial period.

**Translational relevance:**

Measurement of GCL thickness would be a useful indicator of CRAO progression in a clinical trial of putative inhibitors.

## Introduction

**Fig 1:**
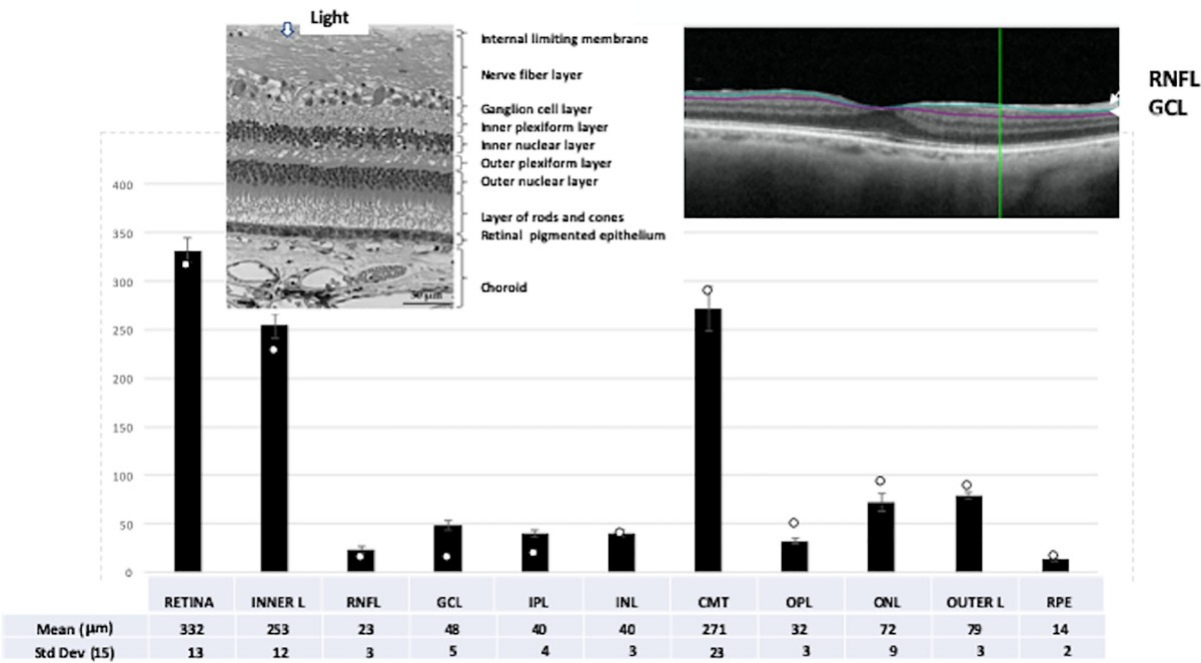
Non-CRAO eyes: Summary of thickness values for each retinal layer with standard deviation bars from 15 patients in the entire cohort of non-affected eyes. For comparison, the white markers on the graph show thickness values from one CRAO eye at 38 days, showing the specific effect on the inner, but not outer layers. (Right image: OCT of a non-CRAO eye showing auto-segmenation of RNFL and GCL. Left image: Histology of retina, enhanced from [22] under Creative Commons).

Occlusion in the central retinal artery (CRAO) is a medical emergency due to the sudden, marked, and often permanent loss of vision in the affected eye [1]. The end organ ischemia produced in retina principally affects the inner layers because the occluded central retinal artery is the main blood supply. From inside to out, the inner layers consist of the axonal retinal nerve fiber layer (RNFL), the somatic ganglion cell layer (GCL), inner plexiform layer (INPL), and the inner nuclear layer (INL) (Fig 1, Upper Left). The long RNFL fibers converge as the optic nerve. The ischemia of the inner layers in CRAO is sometimes mitigated by the presence of a vessel variation, the cilioretinal artery, present in some patients. The sparing effect on loss of vision by the cilioretinal artery occurs in approximately 15% of the population and depends on size of this artery and area of the macula it supplies [2]. The outer layers of eye consist of the outer plexiform layer (OPL), outer nuclear layer (ONL), outer (photoreceptor) layer, and the retinal pigment epithelium (RPE). The outer layers are generally not directly affected in CRAO because they are supplied by the separate, peripheral ciliary artery system that also supplies the choroid and iris.

Characteristics of CRAO depend on the stage. During the early acute phase, observations sometimes include swelling, retinal opacity, whitening and edema of the inner layers and optic nerve head, a cherry red spot in the macula, delayed filling of vessels in the macular arcade, arterial attenuation, boxcarring of the arteries, and pyknosis of the GCL nuclei. Later stage characteristics include further vessel attenuation, atrophy and thinning of the inner retinal layers, and flattening of the foveal depression. Risk factors for CRAO are similar to systemic atherosclerosis and include ipsilateral carotid stenosis, elevated blood lipids, history of heart attack or stroke, diabetes, hypertension, smoking, and older age [2].

Treatment of CRAO is based on quick reperfusion of the retina and restoration of oxygen delivery, with an effective treatment window of within 4–6 hours [3]. Treatments include: manual ocular massage, reduction of intraocular pressure (e.g., IV mannitol), vasodilation (paper bag or carbogen inhalation), and thrombolytic therapy (tissue plasminogen activator) [4]. However, there is no consensus on treatment, and the final visual acuity of 90% of non-arteritic CRAO patients without cilioretinal artery sparing is 20/400 or worse [5].

Our hypothesis is that at least part of the cellular damage to the RNFL and GCL in CRAO is due to activation of the calpain system of calcium-dependent proteases [6, 7]. A calpain inhibitor, SNJ-1945, ameliorated changes in the RNFL and GCL due to hypoxia in monkey retinal transplants. This inhibitor can be administered orally and is currently undergoing human safety trials. Thus, the purposes of the present experiments were: 1) to use segmentation analysis of OCT images to mathematically describe the loss of retinal layers in CRAO patients, and 2) predict the number of patients and days of observation needed for a clinical trial of an inhibitor against CRAO.

## Materials and methods

Using OHSU IRB protocol # 15821 (consent waived), a retrospective, case control study was conducted by computer-aided search in a medical records database for CRAO (ICD10 H43.1) with at least one OCT procedure (CPT: 92134). The research followed the tenets of the Declaration of Helsinki. Appropriate patients and suitable OCT reports were approved by co-author Dr. Thomas Hwang, a clinical retinologist. Layer thickness values from the OCT images stored in e2e format were recorded for the central macula and the mean of the 4 quadrants from the 3 mm ring of the 1, 3, 6 Early Treatment Diabetic Retinopathy Study (ETDRS) grid using Heidelberg Eye Explorer License Manager Control Panel Version 3.1.0.55 [8]. The middle ring was chosen for analysis because it is associated with acute vision yet contains all inner and outer retinal layers. The two groups in this study were CRAO eyes (19 patients) and a control group made up of 15 non-CRAO eyes. Eyes were excluded on the basis of poor layer segmentation. As confirmed by an experienced ophthalmology image analyst, co-author Peter N. Steinkamp), no manual segmentation correction was appropriate, and repeat exams were performed in the follow-up mode. Patient data were best fit in non-linear regression analysis to a decay model:

$$Layer\;thickness=plateau+\left(initial–plateau\right)\;*\;e^{\left(‐k\;*\;DAYS\right)}$$

(k is decay constant) using Prism (GraphPad, ver.8). Tests for homoscedasticity and normality of residuals by D’Agostino and Pearson omnibus K2 were not passed. Therefore, the reference value for goodness fit, R2 (coefficient of determination), was considered biologically significant only if > 0.4.

Sample sizes were determined for various drug efficacies (10%, 20%, 30%, 50%, and 75%) specifying a two tailed t-test with 0.05 significance level and 0.8 as the power of the test [9]. Root mean squared error was used as an estimation of the experimental error when calculating the sample size. Further data at specific time points would be meaningful for better prediction accuracy. The minimum number of patients needed in a clinical trial were predicted using a convenient sample size calculator [9]. For negative control eyes not expected to show rapid thickness changes in the macula, we also analyzed the autosegmented OCT images (n = 23) from seven verified patients with slowly developing hereditary optic neuropthy ranging in age from 11 to 76 years.

## Results

### Demographics

CRAO data were obtained from 19 patients with an average age at onset of 61.2 ± 16.5 years (range 31–82); 63% were male, and 89% were non-Hispanic white patients. CRAO (n = 60) and 15 non-CRAO OCT scans were evaluated by segmentation software, covering a time period from 0 to 951 days from the initiation of CRAO. The number of scans per patient ranged from 1 to 8 depending on recall visits. Approximately 89% of the patients showed severe loss of visual acuity (20/400 to detection of hand motion). Many patients showed co-morbidity factors.

### Retinal segmentation in non-affected control group

CRAO commonly affects one eye. Thus, for T0 controls, we used 15 non-affected contralateral eyes from 19 CRAO patients in this study. (Four CRAO patients had no suitable non-affected contralateral eye data). The mean age of non- affected control patients at 59.6 ± 16.7 (15) years with a 2.0:1 male/female ratio matched well with CRAO patients at 61.2 ± 16.5 (19) years with a 2.2:1 male/female ratio.

**Fig 2:**
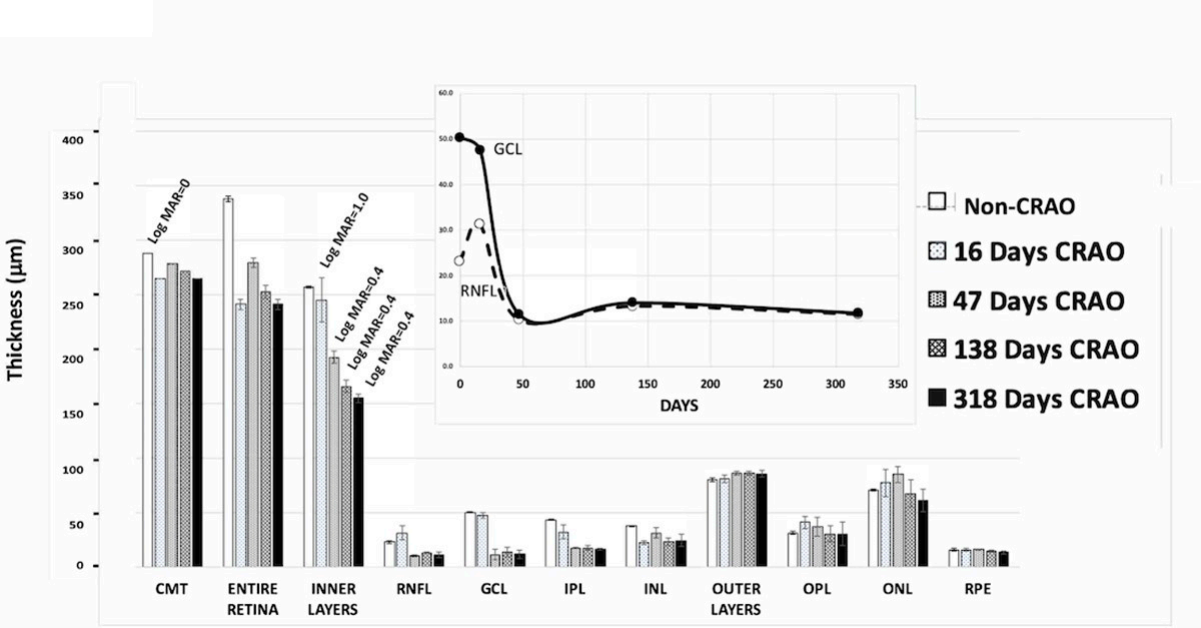
Mean thickness (Y axis, μm) for 11 retinal layers (x-axis) resolved by automated segmentation analysis at increasing days (increasing bar density) after the CRAO. The CRAO for this patient occurred at age 67 and partially recovered. (Insert: Thickness changes in RNFL and GCL over time). The contralateral eye is shown as the clear bar at the far left for each layer. Error bars are ± standard deviation for the mean thickness of the four quadrants of the middle 3 mm ETRS circle.

**Fig 3:**
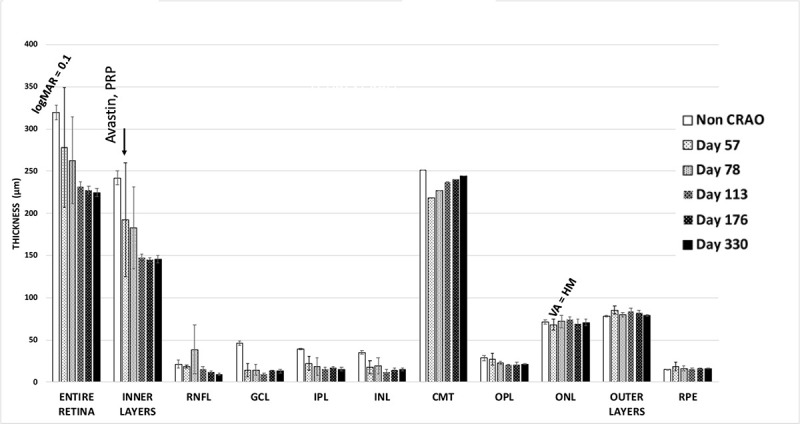
Patient with CRAO occurring at age 72. The patient also had with severe neo-vascular glaucoma that was treated with Avastin and PRP. See legends in Fig 2.

**Fig 4:**
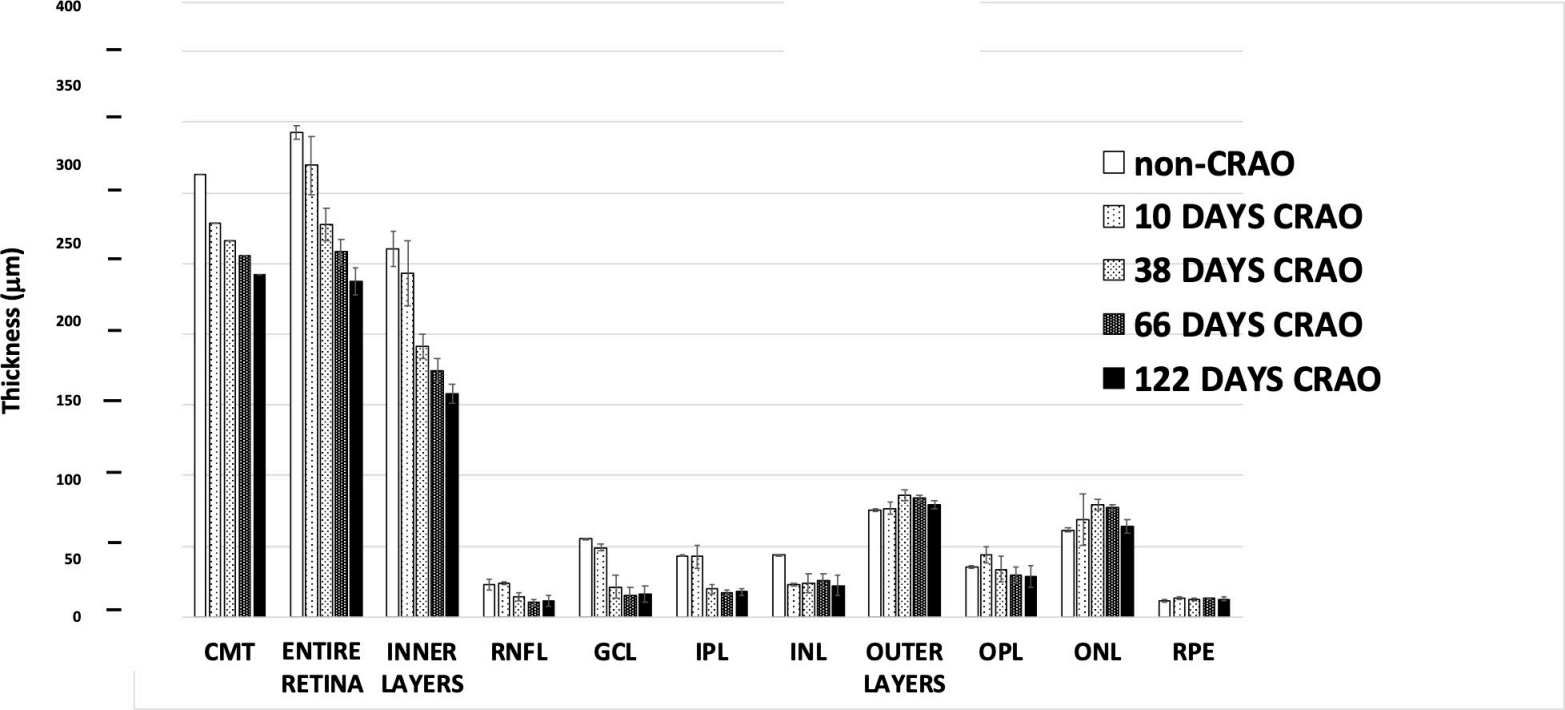
Patient with CRAO occurring at age 71, with embolic stroke of undetermined origin. See legends in Fig 2.

The thickness values in the middle ETDRS ring for the non-CRAO eyes (Fig 1) were similar to literature values [10, 11] for normal patients. Except for CMT, non-CRAO layer thicknesses showed fairly low variability, even though our “normal” patients ranged in age from 31 to 82 years of age and exhibited a number of major co-morbidity eye conditions. The relative stability of the data justified using these non-affected control eyes as the “0” time point when comparing the thickness values after the initial CRAO event in Figs 2–4.

### Retinal layer segmentation in the CRAO group

Affected patients in this study often showed typical signs of CRAO including, marked loss of vision, cherry red spot in macula, swelling of retina at early timepoints, attenuation of vessels, whitening of the macula, and thinning of the inner layers. Three individual patients demonstrating some of the typical changes in layers thicknesses are shown Figs 2–4. The thickness of the RNFL and GCL retinal layers decreased rapidly with time following what appeared to be an exponential decay curve (Fig 2, insert). The most dramatic or substantial change was observed in the inner retina, though the outer retinal layers also exhibited some loss.

### Exponential decay of inner layers with time

**Fig 5:**
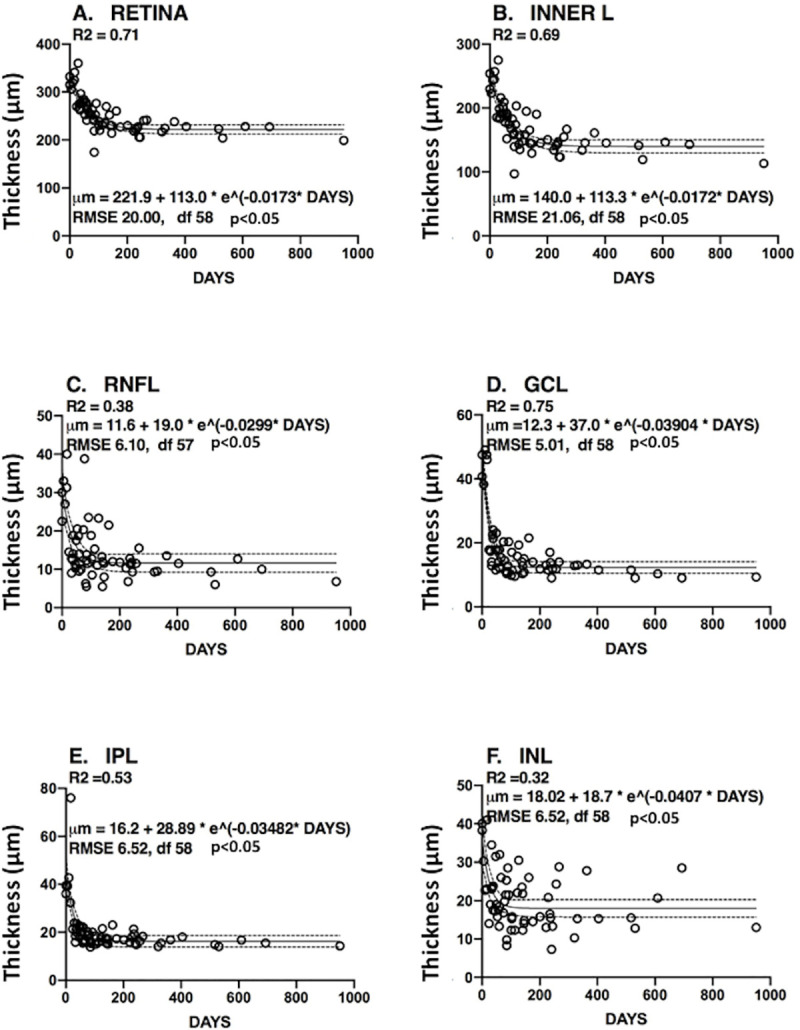
Regression lines (solid) and confidence internals (dashed), showing loss of specific retinal layers during CRAO (Panels A-F include inner layers). The layer-specific exponential decay formula is shown in each panel along with the coefficient of determination (R2). Y-axis is the layer thickness (μm). Open circles are data points from CRAO eyes at individual time points. The abbreviations used for retinal layers are: RETINA = Entire retina. INNER Layers = RNFL + GCL + IPL + INL. RNFL = Retinal nerve fiber layer. GCL = Ganglion cell layer. IPL = Inner plexiform layer. INL = Inner nuclear layer. CMT = Central macular thickness (mean in the 1 mm diameter area of the fovea). OPL = Outer plexiform layer. ONL = Outer nuclear layer. OUTER Layer = Includes photoreceptor elements. RPE = Retinal pigment epithelium.

**Fig 6:**
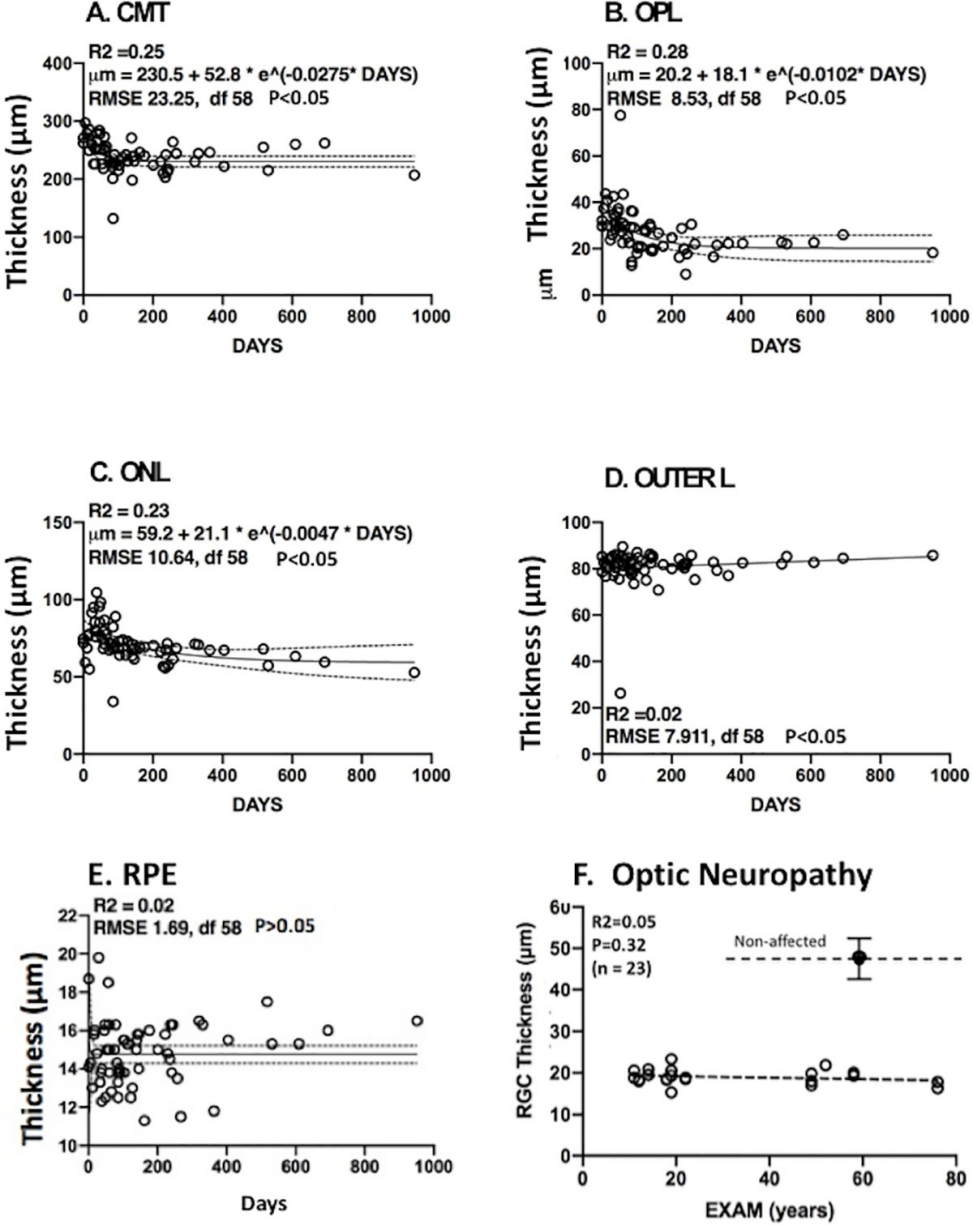
Outer retinal layers during CRAO. See legends in Fig 5.

Changes in thickness of the retina and inner layers in CRAO patients over time fit to the basic exponential model of decay, y = a + b*e(-k*x) (Figs 5 and 6). The fit was especially robust for GCL thickness vs Days (R2 = 0.75, Fig 5D), INNER LAYERS (R2 = 0.69, Fig 5B) and the entire RETINA (R2 = 0.71, Fig 5A). The fit for RNFL was only R2 = 0.38 (Fig 5C). The INL (Fig 5F) showed an R2 = 0.32 with wide scatter, possibly due a secondary blood supply along with the central retinal artery. The outer layers (Fig 6) did not meet our R2 significance threshold and were not further analyzed. As a further negative control for our CRAO studies, RGC changes were measured in hereditary optic neuropathies (Fig 6F). RGC thickness loss (60.5 ± 3.6%) from 7 patients with various with optic neuropathies, were already thinner than in the non-affected eyes in our present studies, indicating that such neuropathies would be difficult to follow in the short term with adult patients.

### Patient sample size and duration of clinical trial

The specific regression formulas for each inner layer shown in each panel of Fig 5 were used to calculate layer thickness at potential clinical trial end points of 30, 60, 90 120 and 180 days (Table 1, column A, “T0 No Drug”). Predicted decreases in thickness due to CRAO after 180 days ranged from– 32 (entire retina) to -74% (GCL) (column B).

**Table 1:** Patients required per group to detect efficacies against CRAO.

	A	B	C	D		E		F		G		H
	No Drug											
DAYS	T0 (um)	(%)	T10	Patients	T20	Patients	T30	Patients	T50	Patients	T75	Patients
**Retina**	332.3											
**30**	289.1	-13.0	293.5	**338**	297.8	**86**	302.1	**39**	310.7	**15**	321.5	**8**
**60**	261.9	-21.2	269.0	**128**	276.0	**33**	283.0	**16**	297.1	**7**	314.7	**4**
**90**	245.7	-26.1	254.4	**85**	263.0	**22**	271.7	**11**	289.0	**5**	310.7	**3**
**120**	236.1	-28.9	245.7	**69**	255.3	**18**	264.9	**9**	284.2	**4**	308.2	**3**
**180**	226.9	-31.7	237.5	**58**	248.0	**16**	258.5	**8**	279.6	**4**	306.0	**3**
**Inner L**	253.8											
**30**	207.6	-18.2	212.3	**329**	216.9	**83**	221.5	**38**	230.7	**15**	242.3	**7**
**60**	180.4	-28.9	187.7	**130**	195.1	**34**	202.4	**16**	217.1	**7**	235.4	**4**
**90**	164.1	-35.3	173.1	**88**	182.1	**23**	191.0	**11**	209.0	**5**	231.4	**3**
**120**	154.4	-39.2	164.3	**72**	174.3	**19**	184.2	**9**	204.1	**5**	229.0	**3**
**180**	145.1	-42.8	156.0	**60**	166.9	**11**	177.7	**8**	199.5	**4**	226.6	**3**
**RNFL**	22.5											
**30**	19.4	-13.8	19.7	**6037**	20.0	**1510**	20.3	**672**	20.9	**243**	21.7	**109**
**60**	14.8	-34.2	15.6	**990**	16.3	**249**	17.1	**111**	18.6	**41**	20.6	**19**
**90**	12.9	-42.5	13.9	**638**	14.8	**161**	15.8	**72**	17.7	**27**	20.1	**13**
**120**	12.2	-45.9	13.2	**551**	14.2	**156**	15.3	**62**	17.3	**23**	19.9	**11**
**180**	11.7	-47.9	12.8	**503**	13.9	**127**	15.0	**57**	17.1	**22**	19.8	**11**
**GCL Days**	47.5											
**30**	23.8	-49.9	26.2	**71**	28.5	**19**	30.9	**9**	35.6	**4**	41.6	**3**
**60**	15.9	-66.5	19.0	**40**	22.2	**11**	25.4	**6**	31.7	**3**	39.6	**3**
**90**	13.4	-71.8	16.8	**35**	20.2	**10**	23.6	**5**	30.5	**3**	39.0	**3**
**120**	12.6	-73.5	16.1	**34**	19.6	**10**	23.1	**5**	30.1	**3**	38.8	**3**
**180**	12.3	-74.1	15.8	**33**	19.4	**3**	22.9	**5**	29.9	**3**	38.7	**3**
**IPL**	39.5											
**30**	26.4	-33.1	27.7	**390**	29.0	**99**	30.3	**45**	32.9	**17**	36.2	**8**
**60**	19.8	-49.9	21.8	**174**	23.7	**45**	25.7	**21**	29.7	**8**	34.6	**5**
**90**	17.5	-55.7	19.7	**139**	21.9	**36**	24.1	**17**	28.5	**7**	34.0	**4**
**120**	16.7	-57.7	19.0	**130**	21.2	**34**	23.5	**16**	28.1	**7**	33.8	**4**
**180**	16.3	-58.7	18.6	**125**	20.9	**32**	23.2	**15**	27.9	**7**	33.7	**4**
**INL**	40.1											
**30**	23.5	-41.4	25.2	**235**	26.8	**60**	28.5	**28**	31.8	**11**	36.0	**6**
**60**	19.6	-51.1	21.7	**156**	23.7	**40**	25.8	**19**	29.9	**8**	35.0	**4**
**90**	18.5	-53.9	20.7	**140**	22.8	**36**	25.0	**17**	29.3	**7**	34.7	**4**
**120**	18.2	-54.6	20.4	**136**	22.5	**35**	24.7	**16**	29.1	**7**	34.6	**4**
**180**	18.0	-55.2	20.2	**134**	22.4	**35**	24.7	**16**	29.1	**7**	34.6	**4**

*T0 through T75 indicate layer thicknesses (μm) at 0 (no drug), 10, 20, 30, 50 and 75% drug efficacies.

Predicted layer thickness at day x = (T0—Tx) times decimal drug efficacy + Tx.

Sample size analysis was also performed to predict the minimum number of CRAO patients needed to detect prevention of thickness loss at various drug efficacies: 10% (Table 1, column D), 20% (column E), 30% (column F), and 50% (column (G), and 75% (column H). For example, 19 patients per group (treated and non-treated) would be required to detect 20% efficacy in retaining the GCL layer in a 30-day trial (box). As expected, as drug efficacy increased, fewer patients would be needed (columns E, F, G & H). Composite measurements, such as the entire retina thickness (86 patients per group, 20% efficacy, 30-day trial) and inner layer thickness (83 patients per group, 20% efficacy, 30-day trial) could also be used as more general biologic markers to follow CRAO treatments.

## Discusssion

### GCL thickness as a marker for following CRAO

A conclusion of the studies above was that measurement of GCL thickness would be a useful indicator of CRAO progression in a clinical trial of potential drugs to ameliorate retinal damage. Data supporting this conclusion include: 1) Previous investigators found OCT measurements in specific layers of the macula to be useful for following the progression of CRAO [12], as well as in other ocular diseases affecting the macula including age-related macular degeneration, diabetic retinopathy, retinal venous occlusion, and even glaucoma [11, 13]. 2) The fit (R2 = 0.75) for negative exponential decay for GCL during the acute and chronic phases of our CRAO cases was good (Fig 5D). A somewhat similar loss of total thickness in the perimacular region versus days was observed, which was described as linear in the chronic phase [14]. A more recent study [15] of 134 CRAO eyes found that 78% showed macular edema at initial baseline, and that 99% showed retinal thinning at final examination. 3) Poor definition of the RNFL and GCL in the peri/para macular region was noted previously [16]. Normal RNFL is especially thin (~ 23 microns) near the fovea, possibly causing greater error when measuring the thinner RNFL in our studies. 4) Thickness measurements for 11 layers and zones in eyes not affected by CRAO eyes (Fig 1) were similar to those in a normative data base based on 2000 eyes [10]. This validated the automated segmentation analysis used in the present study.

### Establishment of clinical trial parameters

The second contribution of the current studies was that clinical trials to test inhibitors were possible with the limited supply of CRAO patients. Even with a large data base at our institution, the number of CRAO patients with available OCT scans was limited, compared to more numerous central retinal vein occlusion/branch retinal vein occlusions. Using OCT GCL layer thickness monitoring, our data indicated that we could theoretically detect 20% efficacy of an inhibitor with 19 CRAO and 19 non-CRAO (collateral eye) patients in a 30-day trial (Table 1, column E). Stronger drug efficacies further reduced the number of required patients (columns F, G & H).

Using SNJ-1945 in such a human trial would further test our hypothesis that at least part of the inner layer thinning in CRAO is due calpain-induced proteolysis [6, 17]. This is based on a series of observations over the last 20 years. For example, the calpain system is present in nearly all animal tissues [7], including non-human primate [6] and human [18] retina. After central retinal artery occlusion and reperfusion in rats, retinal calcium levels increased, proteolysis was observed, and retinal ischemia caused the GCL to slough off [19]. Acute ocular hypertension in rats caused decreased thickness in the IPL and INL, the number of cells in the GCL decreased, TUNEL staining in the INL increased, and a-waves in the ERGs were temporarily decreased [20]. Non-human primate explants cultured under hypoxia showed calpain specific breakdown products in the RNFL followed by TUNEL-staining later in the GCL layer. Importantly, SNJ-1945 ameliorated these changes. In cultured human retinas, hypoxia caused calpain activation and proteolysis; changes that were also ameliorated by SNJ-1945 [18]. The mechanism suggested by these observations for CRAO is that occlusion causes ischemia in the RNFL, energy production by the mitochondria decreases, calcium export from the RNFL is compromised, increased intracellular calcium activates calpains, proteins such as α-spectrin in the axonal RNFL are broken down by calpains, and calpain also degrades its natural inhibitor (calpastatin). Pro-apoptotic signal activation in the axons results in apoptotic thinning of the GCL. Although less specific for this calpain/SNJ-1945 hypothesis, our data in Table 1 showed total retina thickness and composite inner layer thickness measurements could also be used as surrogate markers for CRAO.

### Limitations

To provide a convenient procedure for clinical trials, patients were excluded only if auto-segmentation errors were detected, and manual segmentation was not perfromed. Also, since only a limited number of patients with OCT scans were available, patients in this study showed a wide age range and number of OCT scans; and many co-morbidity factors, concomitant drug and treatment plans, and numerous surgeries and diseases were present. This increased the variability of the data, but represents the practical realities likely to be encountered in finding adequate number of CRAO patients.

Another consideration not addressed in the present study was visual acuity. Thirteen (13) of the 19 patients in the present study showed marked vision loss at CF or worse. Critically, it remains to be tested if vision would be improved by partially preventing loss of retinal GCL and RNFL. If ischemic conditions were of significant duration or if still existed due to the no-reflow phenomenon [21], vision may not be improved by one inhibitor alone. The clinical trial protocol suggested by our study may be applicable for numerous types of CRAO treatment agents. For example, a calpain inhibitor might be useful in conjunction with other treatments that quickly correct ischemic conditions in CRAO.

## Supporting information

S1 Data (XLSX)
